# The Causal Role of Mitochondrial Dynamics in Regulating Insulin Resistance in Diabetes: Link through Mitochondrial Reactive Oxygen Species

**DOI:** 10.1155/2018/7514383

**Published:** 2018-09-30

**Authors:** Hung-Yu Lin, Shao-Wen Weng, Yen-Hsiang Chang, Yu-Jih Su, Chih-Min Chang, Chia-Jen Tsai, Feng-Chih Shen, Jiin-Haur Chuang, Tsu-Kung Lin, Chia-Wei Liou, Ching-Yi Lin, Pei-Wen Wang

**Affiliations:** ^1^Department of Internal Medicine, Kaohsiung Chang Gung Memorial Hospital and Chang Gung University College of Medicine, Kaohsiung 833, Taiwan; ^2^Lee's Endocrinology Clinic, Pingtung, Taiwan; ^3^Department of Nuclear Medicine, Kaohsiung Chang Gung Memorial Hospital and Chang Gung University College of Medicine, Kaohsiung 833, Taiwan; ^4^Department of Surgery, Kaohsiung Chang Gung Memorial Hospital and Chang Gung University College of Medicine, Kaohsiung 833, Taiwan; ^5^Department of Neurology, Kaohsiung Chang Gung Memorial Hospital and Chang Gung University College of Medicine, Kaohsiung 833, Taiwan

## Abstract

**Background:**

Mitochondrial dynamics (mtDYN) has been proposed as a bridge between mitochondrial dysfunction and insulin resistance (IR), which is involved in the pathogenesis of type 2 diabetes (T2D). Our previous study has identified that mitochondrial DNA (mtDNA) haplogroup B4 is a T2D-susceptible genotype. Using transmitochondrial cybrid model, we have confirmed that haplogroup B4 contributes to cellular IR as well as a profission mtDYN, which can be reversed by antioxidant treatment. However, the causal relationship between mtDYN and cellular IR pertaining to T2D-susceptible haplogroup B4 remains unanswered.

**Methods:**

To dissect the mechanisms between mtDYN and IR, knockdown or overexpression of MFN1, MFN2, DRP1, and FIS1 was performed using cybrid B4. We then examined the mitochondrial network and mitochondrial oxidative stress (mtROS) as well as insulin signaling IRS-AKT pathway and glucose transporters (GLUT) translocation to plasma membrane stimulated by insulin. We employed Drp1 inhibitor, mdivi-1, to interfere with endogenous expression of fission to validate the pharmacological effects on IR.

**Results:**

Overexpression of MFN1 or MFN2 increased mitochondrial network and reduced mtROS, while knockdown had an opposing effect. In contrast, overexpression of DRP1 or FIS1 decreased mitochondrial network and increased mtROS, while knockdown had an opposing effect. Concomitant with the enhanced mitochondrial network, activation of the IRS1-AKT pathway and GLUT translocation stimulated by insulin were improved. On the contrary, suppression of mitochondrial network caused a reduction of the IRS1-AKT pathway and GLUT translocation stimulated by insulin. Pharmacologically inhibiting mitochondrial fission by the Drp1 inhibitor, mdivi-1, also rescued mitochondrial network, reduced mtROS, and improved insulin signaling of diabetes-susceptible cybrid cells.

**Conclusion:**

Our results discovered the causal role of mtDYN proteins in regulating IR resulted from diabetes-susceptible mitochondrial haplogroup. The existence of a bidirectional interaction between mtDYN and mtROS plays an important role. Direct intervention to reverse profission in mtDYN provides a novel therapeutic strategy for IR and T2D.

## 1. Introduction

The mitochondrion, an organelle responsible for the production of ATP, plays a central role in cellular metabolism [1]. Mitochondria form a complex dynamic network that continuously undergoes fusion and fission events, known as mitochondrial dynamics (mtDYN). Mitochondrial dynamics is a quality control system critical for maintaining the mitochondrial population and relevant to the stability of mitochondrial DNA (mtDNA), respiratory capacity, and cell response to stress [2]. Quality control might not be the only task carried out by mtDYN. Recent studies link mtDYN to the balance between energy demand and nutrient supply [3]. Several lines of evidence revealed alterations in mtDYN in insulin-resistant states and type 2 diabetes (T2D) in humans and in animal models [4–10]. Therefore, mtDYN have been implicated in the development of insulin resistance (IR) [9, 11, 12] and the pathogenesis of T2D [7, 13, 14].

In our previous work, we have firstly recognized that subjects harboring mitochondrial haplogroup B4, consisting of 16189T⟶C transition and 10398A allele, are diabetes-susceptible risk factor [15]. By the construction of transmitochondrial cybrid cell, we verified the impact of haplogroup B4 on cellular IR [16] as well as the imbalance of mtDYN toward a profission state [17]. Further, antioxidant treatment causes concomitance of the profusion manner of mtDYN and improved IR in cybrid B4 [17]. However, the causal relationship of both physiological events has not been clarified. To address whether mtDYN plays a causal role in regulating cellular IR and serves as a potential therapeutic target, we have bidirectionally manipulated the expression of dynamic proteins, including *MFN1*, *MFN2*, *DRP1*, and *FIS1* in cybrid cell harboring diabetes-susceptible haplogroup B4. We also employed Drp1 inhibitor, mdivi-1, to interfere with endogenous expression of fission to validate the pharmacological effects on IR.

## 2. Materials and Methods

### 2.1. Cell Culture and Cybrid Generation

Cells were cultured using Dulbecco's modified eagle's medium (DMEM, high glucose, Gibco, Carlsbad, CA, USA) supplemented with 10% heat-inactivated fetal bovine serum (FBS; Gibco, Carlsbad, CA, USA) at 37°C in 5% CO_2_. The generation of transmitochondrial cybrid was previously described [18]. Briefly, cybrids were generated by fusing 143B-*ρ*^0^ cells with human platelets in the absence of pyruvate and uridine. Platelets were isolated from a volunteer subject harboring mtDNA haplogroup B4 and fused with *ρ*^0^ cells in the presence of polyethylene glycol 1500 (50% *w*/*v*; Roche, Nutley, NJ, USA). This study protocol and written informed content were reviewed and approved by the Institutional Review Board of Chang Gung Memorial Hospital (CGMH; IRB number 101-1620A3).

### 2.2. Experimental Procedure

Cells were starved of FBS for 16 h, followed by transfection of plasmid/siRNA or mdivi-1 treatment. Then, cells were stimulated with insulin (Sigma-Aldrich, St. Louis, MO, USA) at 0, 0.1, or 1 *μ*M for 1 h and then collected for experimental assays. Gene overexpression and knockdown of Mfn1, Mfn2, Drp1, and Fis1 were performed using Lipofectamine® 2000 (Invitrogen; Thermo Fisher Scientific, Inc., Waltham, MA, USA). Transfection of plasmid (RG207184; RG202218; RG202046; RG202560; Origene Technologies, Inc., Rockville, MD, USA) and siRNA (sc-43927; sc-43928; sc-43732; sc-60643; Santa Cruz Biotechnology, Santa Cruz, CA) spanned 24 and 48 h, respectively. Mock control of gene overexpression and siRNA was GFP-expression vector (OriGene-ORIPS100010, Origene Technologies, Inc., Rockville, MD, USA) and dsRNA with scramble sequence (sc-37007; Santa Cruz Biotechnology, Santa Cruz, CA), respectively.

### 2.3. Mitochondrial Morphology

Mitochondria were visualized using mitochondrial-targeting fluorescent protein cox4-DsRed, which is a kind gift from Dr. David Chan (California Institute of Technology, Pasadena, CA 91125, USA). The MicroP algorithm categorized mitochondrial morphology into six types: small globe (blue), large globe (yellow), simple tube (green), twisted tube (orange), donut (red), and branching tube (purple). *N* = 75–400 mitochondria from 15–30 cells and three independent experiments.

### 2.4. Subfractionation of Cell Membrane

The Thermo Scientific Subcellular Protein Fractionation Kit for Cultured Cells (Thermo Fisher Scientific Inc., Rockford, IL, USA) was used to stepwise separate cytoplasmic, membrane, nuclear soluble, chromatin-bound, and cytoskeletal protein extracts from the cultured cybrid B4 cells. Briefly, cell pellet (2 × 10^6^ cells in 20 *μ*L packed cell volume) was incubated with 200 *μ*L CEB at 4°C for 10 minutes with gentle mixing and then centrifuged at 500 × g for 5 minutes. The supernatant was immediately transferred to a clean prechilled tube on ice to get the cytoplasmic extract. The pellet was mixed with ice-cold MEB, vortexed the tube for 5 seconds, incubated at 4°C for 10 minutes with gentle mixing, and then centrifuged at 3000 × g for 5 minutes. The supernatant was transferred to a clean prechilled tube on ice to get the membrane extract. The nuclear soluble, chromatin-bound, and cytoskeletal protein extracts were separated stepwise according to the instruction and stored at −80°C, and the cytoplasmic and membrane extracts were maintained on ice for same-day use.

### 2.5. Detection of Mitochondrial Reactive Oxygen Species

The levels of mitochondrial superoxide (O_2_^·−^) produced in the cells were quantified using a MitoSOX Red kit (Molecular Probes, Invitrogen), which comprises a redox-sensitive dye composed of hydroethidine linked by a hexyl carbon chain to a triphenylphosphonium group, which was used to target the mitochondrial matrix due to the negative membrane potential across the inner mitochondrial membrane. The cells were plated at a density of 8 × 10^4^ cells per well in 12-well plates (Nunc, Denmark) with a medium containing 25 mM glucose. MitoSOX Red was added at a final concentration of 1.0 *μ*M in HBSS (Gibco BRL, USA). The cells were then incubated for 10 min at 37°C, and then they were harvested and washed twice with PBS. They were fixed in 4% paraformaldehyde and mounted in Fluoromount media (Sigma-Aldrich Co. LLC), which assisted in the visualization of the slides under a fluorescence microscope (Leica, Wetzlar, Germany). The average fluorescence intensity was quantitatively determined using ImageJ by counting 50–100 cells per field of view, five representative fields per experimental group, and three independent experiments.

### 2.6. Western Blotting

The cells were plated at a density of 2 × 10^6^ cells per well in 6-well plates (Nunc, Denmark). Following an overnight incubation, the cells were serum-starved for 16 h, after which they were treated with a medium containing 25 mM glucose and stimulated with 0, 0.1, and 1.0 *μ*M insulin for 30 min. The cells were harvested, after which their protein extract was isolated using a buffer containing 150 mM NaCl, 50 mM HEPES pH 7, 1% Triton X-100, 10% glycerol, 1.5 mM MgCl_2_, 1 mM EGTA, and a protease inhibitor. The proteins were separated via SDS-PAGE by using an 8–10% polyacrylamide gel, and then they were transferred onto a polyvinylidene fluoride (PVDF) membrane (Millipore) by using a blotting apparatus. The membrane was blocked using 5% milk in TBS-T for 1 h at room temperature and then incubated overnight at 4°C with antibodies against anti-GLUT1 (1 : 1000 dilution from Santa Cruz Biotechnology), anti-GLUT4 (1 : 1000 dilution from Santa Cruz Biotechnology), anti-pIRS1 (Y896) (1 : 1000 dilution from Epitomics, Inc.), anti-IRS1 (1 : 2000 dilution from Merck Millipore), p-Akt (S473) (1 : 1000 dilution from Santa Cruz Biotechnology), anti-Akt (1 : 1000 dilution from Santa Cruz Biotechnology), and anti-*β*-actin (1 : 50000 dilution from Merck Millipore). Further, following conjugation of the secondary antibody with HRP for 60 min, the signals on the membrane were detected using ECL-plus luminal solution (Advansta, USA) and exposed to an X-ray film for autoradiogram.

### 2.7. Statistical Analysis

The database was created using Microsoft Excel and plotted using GraphPad Prism software programs. The results were expressed as mean ± standard error (SE). Student's *t*-test was used to compare groups, whereas a one-way analysis of variance was used when more than two groups were compared. A *P* value of less than 0.05 was considered statistically significant. The experiments were conducted at least three times to verify reproducibility.

## 3. Results

### 3.1. Mitochondrial Network Can Be Remodeled by Manipulating Mitochondrial Dynamic Genes in Diabetes-Susceptible Cybrid Cell

**Figure 1:**
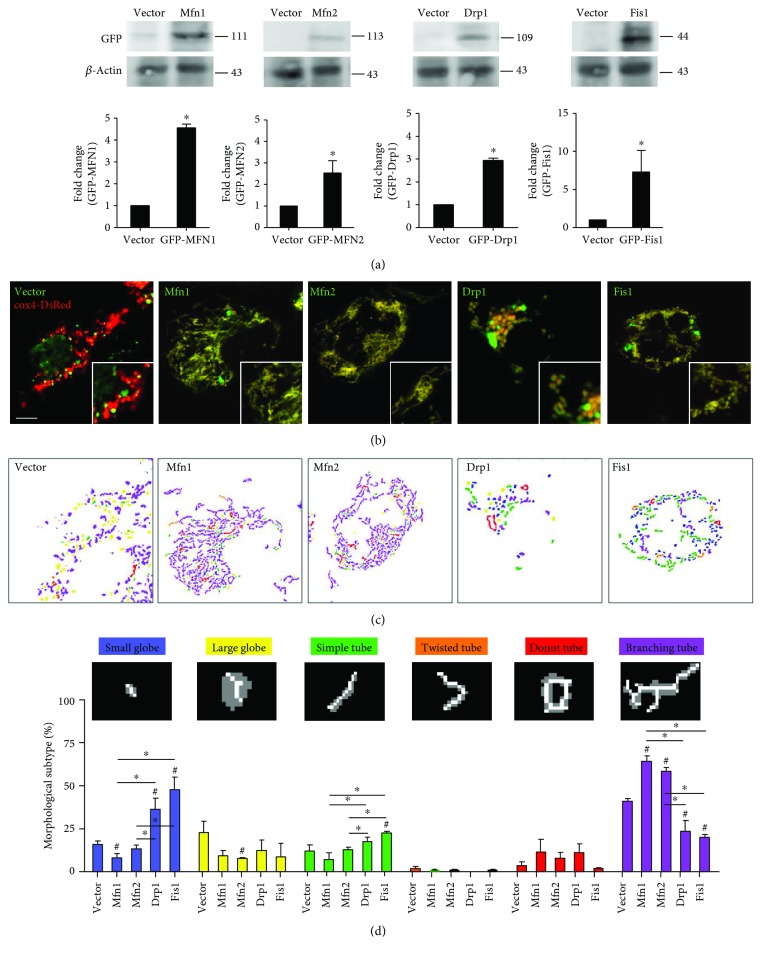
Overexpression of Mfn1/Mfn2 and Drp1/Fis1 promotes mitochondrial fusion and fission in DM cybrid, respectively. Gene overexpression was conducted by the transfecting plasmid. GFP-expressing plasmid (vector) was used as vector control. (a) Abundance of dynamic proteins Mfn1, Mfn2, Drp1, and Fis1 was determined using Western blotting. *β*-Actin served as loading control. (b) Mitochondrial morphology was visualized by transfecting cox4-DsRed (red fluorescence) in DM cybrid expressing GFP alone or GFP-tagged mitochondrial dynamic proteins (green fluorescence). An enlarged segment of each image was shown by a lower right square. (c) The MicroP algorithm categorized mitochondrial morphology into six types: small globe (blue), large globe (yellow), simple tube (green), twisted tube (orange), donut (red), and branching tube (purple). *N* = 75–400 mitochondria from 15–30 cells and three independent experiments.

**Figure 2:**
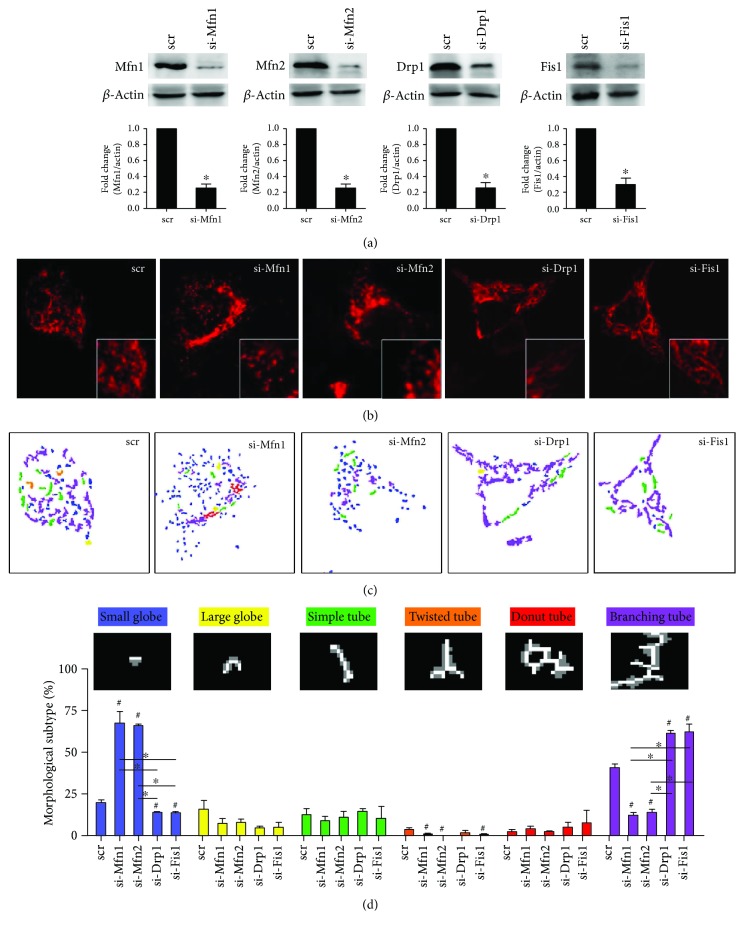
Knockdown of Mfn1/Mfn2 and Drp1/Fis1 reduces mitochondrial fusion and fission in DM cybrid, respectively. siRNA transfection was performed to knockdown target gene expression. Scramble dsRNA (scr) was used as siRNA negative control. (a) Abundance of dynamic proteins Mfn1, Mfn2, Drp1, and Fis1 was determined using Western blotting. *β*-Actin served as loading control. (b) Mitochondrial morphology was visualized by transfecting cox4-DsRed (red fluorescence). An enlarged segment of each image was shown by a lower right square. (c) The MicroP algorithm categorized mitochondrial morphology into six types: small globe (blue), large globe (yellow), simple tube (green), twisted tube (orange), donut (red), and branching tube (purple). *N* = 75–400 mitochondria from 15–30 cells and three independent experiments.

To examine the causal role of mitochondrial dynamics in insulin resistance relevant to mtDNA variants, we established cybrid cell harboring diabetes mellitus- (DM-) susceptible mtDNA haplogroup B4 (thereafter DM cybrid) [18] and overexpressed Mfn1, Mfn2, Drp1, and Fis1 in cybrid B4. Overexpression significantly increased mitochondrial dynamic protein abundance (Figure 1(a)). As mitochondrial dynamic proteins govern fusion/fission manner of mitochondria [2], we thus visualized mitochondrial network by transfecting mitochondrial-targeted fluorescent protein cox4-DsRed and quantifies mitochondrial morphology. Overexpression of fusion-related Mfn1 and Mfn2 resulted in an increased tubular network of mitochondria and reduced fragmentation of mitochondria, whereas overexpression of fission-related Drp1 and Fis1 led to a reversed manner (Figures 1(b)–1(d)). Furthermore, we employed siRNA to interfere with endogenous expression of Mfn1, Mfn2, Drp1, and Fis1 to validate their role in the manner of mitochondrial dynamics. The abundance of Mfn1, Mfn2, Drp1, and Fis1 were significantly reduced by siRNA (Figure 2(a)). Knockdown of Mfn1/Mfn2 showed predominantly fragmental mitochondria, while knockdown of Drp1/Fis1 presented mainly networking mitochondria (Figures 2(b)–2(d)). These results demonstrate that manipulating mitochondrial dynamic genes significantly alter mitochondrial network in DM cybrid.

### 3.2. Mfn1 and Mfn2 Ameliorate Insulin Resistance of Diabetes-Susceptible Cybrid Cell

**Figure 3:**
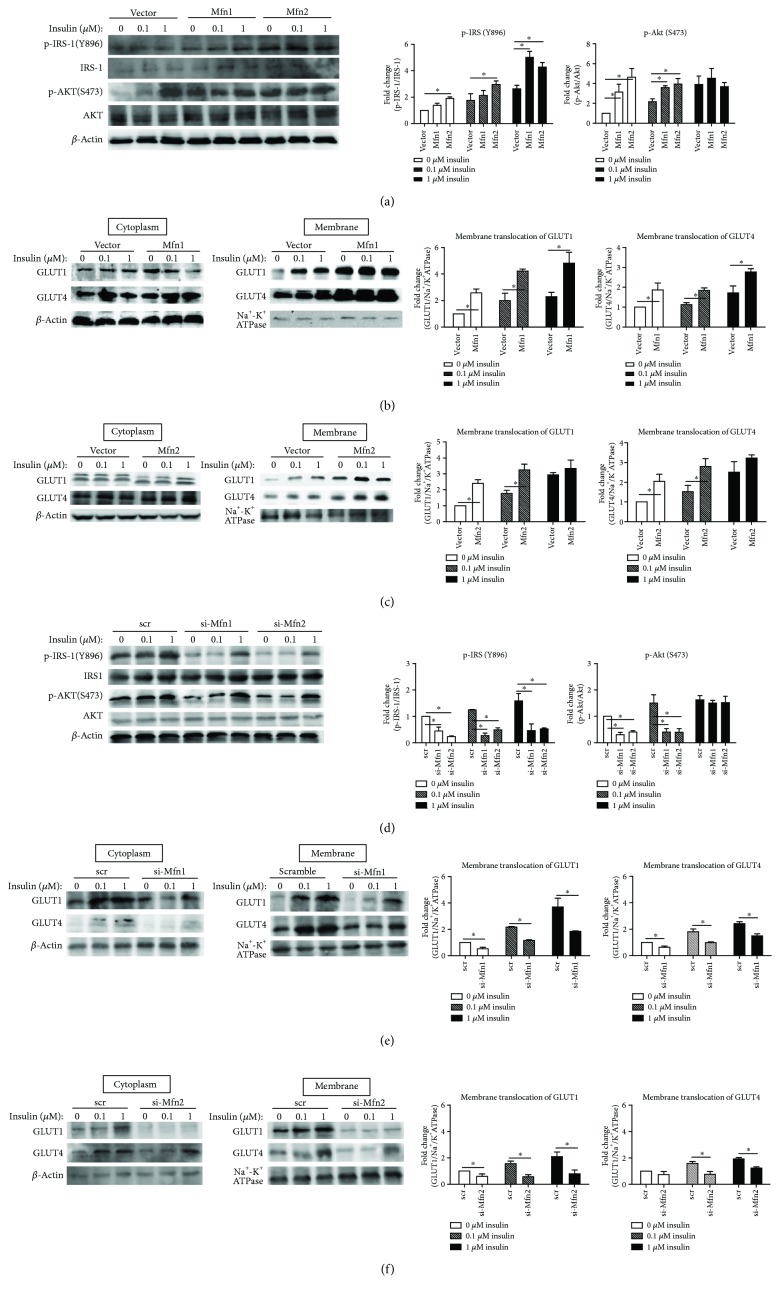
Both Mfn1 and Mfn2 ameliorate cellular insulin resistance. Cybrid B4 was starved of FBS for 16 h, transfected with plasmid/siRNA for 24/48 h, and then treated with 0, 0.1, or 1 *μ*M insulin for 1 h. (a, d) p-IRS1-1(Y896), IRS-1, p-AKT(S473), and AKT were determined using Western blotting. *β*-Actin served as loading control. (b, c, e, f) Abundance of GLUT1 and GLUT4 in cytoplasm and membrane subfractionation was probed using Western blotting. *β*-Actin and Na^+^-K^+^ATPase served as loading control of cytoplasm and membrane fraction. The quantitative result (mean ± SEM) was calculated from at least three independent experiments. ^∗^*p* < 0.05.

As shown in Figure 3(a), the level of insulin-induced activation of IRS-1 phosphorylation of Tyr-896 was found to be increased significantly after overexpression of fusion-related proteins (Mfn1/Mfn2). Overexpression of Mfn2 showed increased p-IRS1 in basal and insulin-treated cells, while Mfn1 increased p-IRS1 in insulin-treated cells only (1 *μ*M). Furthermore, the Akt Ser-473 phosphorylation, which served as a downstream regulator of the PI3 kinase pathway, was also increased significantly after overexpression of fusion-related proteins (Mfn1/Mfn2) in DM-susceptible cybrids. This trend was observed both in basal or after insulin (0, 0.1 *μ*M) treatment. The GLUT1 and GLUT4 translocation to the plasma membrane in DM-susceptible cybrids increased significantly after overexpression of fusion-related proteins (Mfn1/Mfn2) in basal and insulin-treated cells (Figures 3(b) and 3(c)). In contrast, knockdown of fusion-related molecules (Mfn1/Mfn2) significantly decreased the level of basal and insulin-induced activation of IRS-1 and Akt phosphorylation (Figure 3(d)). Furthermore, knockdown of fusion-related molecules decreased the GLUT1/GLUT4 translocation to the plasma membrane in DM-susceptible cybrids (Figures 3(e) and 3(f)). In Mfn1 overexpression cybrids, the trend was both in basal and insulin-treated cells, while the trend in Mfn2 cybrids was in insulin-treated cells only.

### 3.3. Drp1 and Fis1 Deteriorate Insulin Resistance of Diabetes-Susceptible Cybrid Cell

**Figure 4:**
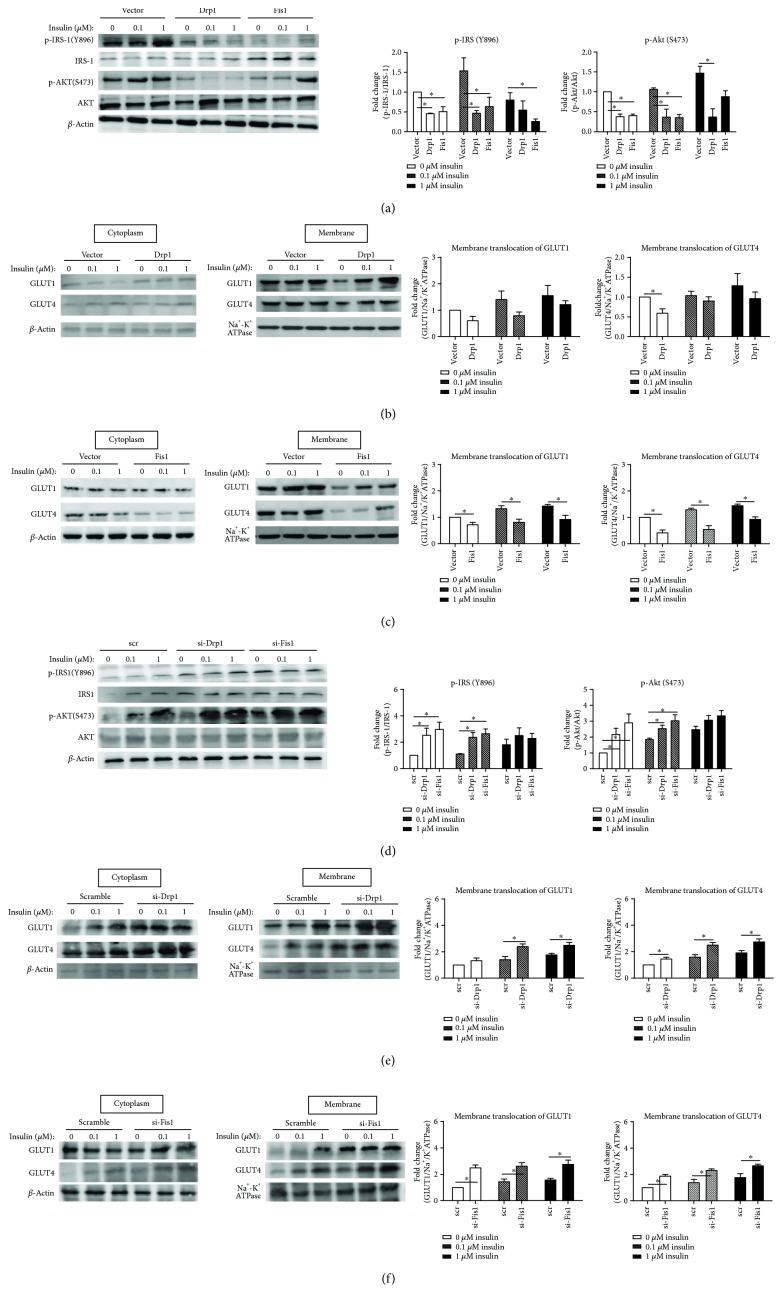
Both Drp1 and Fis1 deteriorate cellular insulin resistance. Cybrid B4 was starved of FBS for 16 h, transfected with plasmid/siRNA for 24/48 h, and then treated with 0, 0.1, or 1 *μ*M insulin for 1 h. (a, d) p-IRS1-1(Y896), IRS-1, p-AKT(S473), and AKT were determined using Western blotting. *β*-Actin served as loading control. (b, c, e, f) Abundance of GLUT1 and GLUT4 in cytoplasm and membrane subfraction at ion was probed using Western blotting. *β*-Actin and Na^+^-K^+^ATPase served as loading control of cytoplasm and membrane fraction. The quantitative result (mean ± SEM) was calculated from at least three independent experiments. ^∗^*p* < 0.05.

As shown in Figure 4(a), the level of both basal and insulin-induced activation of IRS-1 phosphorylation of Tyr-896 was found to be decreased significantly after overexpression of fission-related proteins (Drp1/Fis1). The Akt Ser-473 phosphorylation was also decreased significantly after overexpression of fission-related proteins (Drp1/Fis1) both in basal and after insulin treatment. The GLUT1 and GLUT4 translocation to plasma membrane decreased significantly after overexpression of Fis1 proteins in both basal and insulin-treated cells (Figure 4(c)), while only GLUT4 translocation decreased in basal condition after overexpression of Drp1 proteins (Figure 4(b)). In contrast, knockdown of fission-related molecules (Drp1/Fis1) ameliorate insulin resistance of diabetes-susceptible cybrid cells. Knockdown of Drp1/Fis1 significantly increased the level of basal and insulin-induced activation of IRS-1 and Akt phosphorylation (Figure 4(d)). Furthermore, knockdown of fission-related molecules increased the GLUT1/GLUT4 translocation to the plasma membrane in DM-susceptible cybrids (Figures 4(e) and 4(f)).

### 3.4. Pharmacologically Inhibiting Mitochondrial Fission Also Improves Insulin Resistance of Diabetes-Susceptible Cybrid Cell

**Figure 5:**
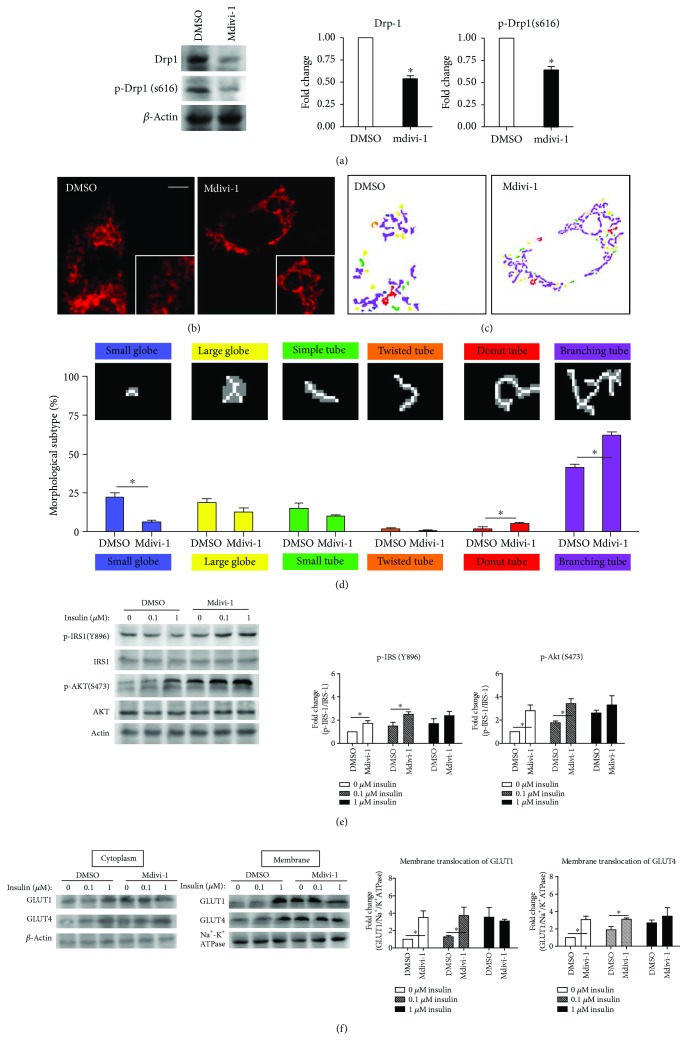
Insulin resistance of cybrid B4 was improved by mdivi-1, which suppresses mitochondrial fission and promotes fusion. 10 *μ*M mdivi-1 was used to inhibit mitochondrial fission. For probing cellular insulin resistance, cybrid B4 was starved of FBS for 16 h, then incubated with mdivi-1 for 24 h, and finally treated with 0, 0.1, or 1 *μ*M insulin for 1 h. (a) The action of Drp1 inhibitor, mdivi-1, was determined using level of p-Drp1(S616) and Drp-1. *β*-Actin served as loading control. (b) Mitochondrial morphology was visualized by transfecting cox4-DsRed (red fluorescence). An enlarged segment of each image was shown by a lower right square. (c-d) The MicroP algorithm categorized mitochondrial morphology into six types: small globe (blue), large globe (yellow), simple tube (green), twisted tube (orange), donut (red), and branching tube (purple). *N* = 75–400 mitochondria from 15–30 cells and three independent experiments. (e) p-IRS1-1(Y896), IRS-1, p-AKT(S473), and AKT were determined using Western blotting. *β*-Actin served as loading control. (f) Abundance of GLUT1 and GLUT4 in cytoplasm and membrane subfractionation was probed using Western blotting. *β*-Actin and Na^+^-K^+^ATPase served as loading control of cytoplasm and membrane fraction. The quantitative result (mean ± SEM) of Western blotting was calculated from at least three independent experiments. ^∗^*p* < 0.05.

We employed Drp1 inhibitor, mdivi-1, to interfere with endogenous expression of fission to validate the pharmacological effects on IR. The abundance of both p-Drp1 and Drp-1 were significantly reduced by mdivi-1 (Figure 5(a)). The mitochondrial network was increased by mdivi-1 resulting in an increased tubular network of mitochondria and reduced fragmentation of mitochondria (Figures 5(b)–5(d)). As to IR, the level of activation of IRS-1 phosphorylation of Tyr-896 and the Akt Ser-473 phosphorylation were increased significantly both in basal and after insulin treatment (0, 0.1 *μ*M) (Figure 5(e)). Furthermore, the GLUT1 and GLUT4 translocation to the plasma membrane in DM-susceptible cybrids was also increased significantly after mdivi-1 treatment both in basal and insulin-treated cells (0, 0.1 *μ*M) (Figure 5(f)).

### 3.5. Dynamic Proteins Modulate Mitochondrial ROS in Cybrid B4

**Figure 6:**
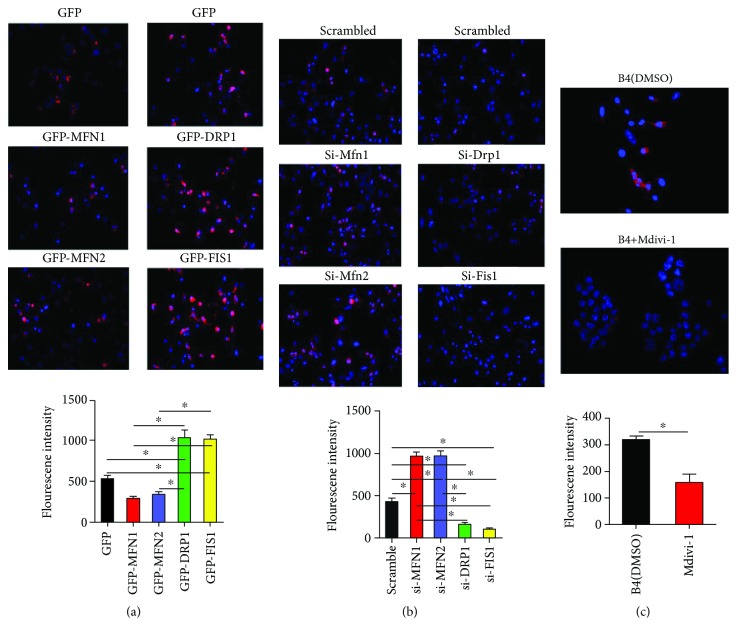
Dynamic proteins modulate mitochondrial ROS in cybrid B4. (a) Cybrid B4 was transfected with plasmid for 24 h to overexpress particular dynamic proteins. (b) Cybrid B4 was transfected with siRNA for 48 h to knockdown particular dynamic proteins. (c) Cybrid B4 was treated with 10 *μ*M mdivi-1 for 24 h to inhibit mitochondrial fission. Mitochondrial ROS production was visualized using MitoSOXRed, which was nuclear counterstained using DAPI. Fluorescent intensity of mitochondrial ROS was quantified using ImageJ. ^∗^*p* < 0.05 between indicated groups.

In our previous work [17], we have verified the imbalance of mtDYN toward a profission state in cybrid B4 and antioxidant treatment reverse it to a profusion manner. In this study, we further tested whether mtDYN may affect the expression of mtROS. As shown in Figure 6(a), overexpression of MFN1 or MFN2 reduced mtROS, while knockdown has an opposing effect (Figure 6(b)). In contrast, overexpression of DRP1 or FIS1 increased mtROS (Figure 6(a)), while knockdown has an opposing effect (Figure 6(b)). Pharmacologically inhibiting mitochondrial fission by the Drp1 inhibitor, mdivi-1 (10 *μ*M, 24 hr), also reduced mtROS (Figure 6(c)).

### 3.6. The Role of Mitochondrial Dynamics in Regulating Insulin Resistance

**Figure 7:**
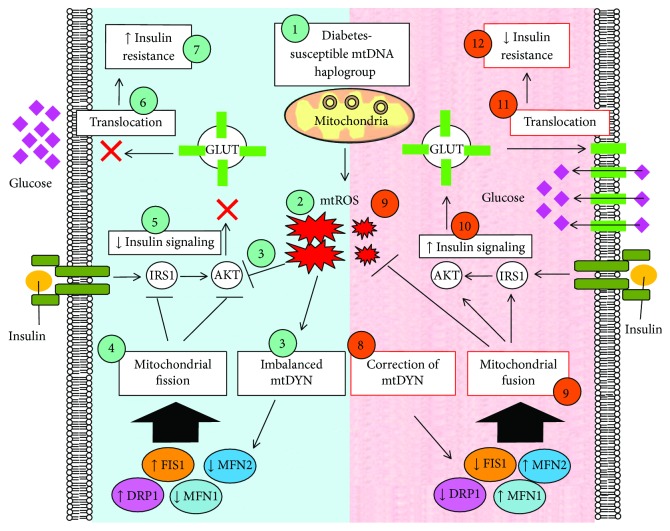
Graphic summary delineates the causal role of mitochondrial dynamics in regulating insulin resistance of diabetes through mtROS. (1-2) Diabetes-susceptible haplogroup B4 demonstrates increased mtROS expression. (3-4) Increased mtROS contributes to insulin resistance and imbalanced profile of mitochondrial dynamics: enhanced fission proteins (Drp1 and Fis1) and reduced fusion proteins (Mfn1 and Mfn2), which ultimately contributes to mitochondrial fission (Kuo et al., 2016; Weng et al., 2013). (5–7) Mitochondrial fission inhibits insulin-activated IRS1-AKT signaling, which subsequently hinders GLUTs from translocating to cellular membrane and leads to insulin resistance. (8) Either genetic manipulation or pharmacological intervention effectively (9) correct mitochondrial dynamics toward a profile of fusion and simultaneously suppresses mtROS expression, which (10) boost insulin-activated IRS1-AKT signaling, and consequently (11) activates GLUTs translocation to cellular membrane and finally (12) improving insulin resistance.

In our previous and current reports with clinical and in vitro studies, we conclude that mitochondrial genotypes and quality control were related to insulin resistance (Figure 7).

## 4. Discussion

In our previous clinical studies, we found that mitochondrial haplogroup B4, consisting of 16189T⟶C transition and 10398A allele, are diabetes-susceptible risk factor [15]. By the construction of transmitochondrial cybrid cell, we verified the impact of haplogroup B4 on cellular IR [16] as well as the imbalance of mitochondrial dynamics [17]. Both defects, IR and profission mtDYN, are reversed by antioxidant treatment [16, 17]. Here, we further demonstrate the causal role of mitochondrial dynamics in IR by manipulating the expressions of dynamic proteins in diabetes-susceptible cybrid cells. Overexpression of fusion-related proteins (Mnf1/Mnf2) and inhibition of fission-related proteins (Drp1/Fis1) enhance mitochondria network and reverse the IR by activating the IRS1-Akt pathway and inducing the GLUT1/GLUT4 translocation to the cell membrane. Pharmacological inhibition of DRP1 by mdivi-1 also reveals improvement of IR, making it probable to be a new era of diabetes treatment.

Mitochondrial dynamics undergo fission and fusion to repair damaged components of the mitochondrial homeostasis. This regulation via the fission process allows for segregation of damaged mitochondria and the fusion process allows the exchange of material between healthy mitochondria [19, 20]. Previous reports in humans and animal models that showed the trend of dynamic proteins toward fission was related to IR/T2D. T2D patients and obese subjects show a reduction in mitofusin 2 (Mfn2) expression in skeletal muscle compared to controls [11], and exercise increases insulin sensitivity in association with a decrease in muscle dynamin-related protein 1 (Drp1) in obese and insulin-resistant humans [21]. As to animal studies, a reduction in the mitochondrial network in the skeletal muscle of obese Zucker rats [6], leptin-deficient (*ob/ob*) mice, and diet-induced obese C57BL/6 mice [9] has been reported. The mitochondria in pancreatic islets from RIP2-Opa1KO mice, which displayed hallmarks of diabetes, were shorter and fragmented with decreased ATP production [8]. Liver-specific ablation of Mfn2 in mice led to numerous metabolic abnormalities, which was characterized by glucose intolerance and enhanced hepatic gluconeogenesis [4]. An increase in Drp1-dependent mitochondrial fission in the muscle was associated with IR in rodents, and inhibition of mitochondrial fission improved the muscle insulin signaling of obese mice [9]. More recently, in the dorsal vagal complex of the high-fat feeding rat, Drp1-dependent mitochondrial fission regulate insulin action and targeting the Drp1-mitochondrial-dependent pathway in the brain may have therapeutic potential in IR [22].

Alterations or mutations in mitochondrial fusion and fission proteins have been identified to be associated with the nutrient availability and energy demand. Mitochondrial fragmentation is caused by nutrient excess, while mitochondrial elongation is induced by nutrient withdrawal in mouse embryonic fibroblast [3, 23, 24]. Furthermore, mitochondria fuse to form elongated mitochondria to maximize ATP production during nutrient shortage, whereas mitochondria tend to form fragmented mitochondrion to prevent overt ATP synthesis during nutrient excess [25]. The molecular events causing diabetes and its complications have been focused on the oxidative stress and chronic inflammation in affected tissue [26]. Recent studies have shown that mitochondria not only generate energy for the physiological process but also emerge as a fundamental hub for innate antiviral immunity [27, 28]. Mitochondrial dynamics may be proposed as a bridge between nutrient excess and chronic inflammation in diabetes.

Before the current study, although the contribution of mitochondrial fusion and fission in IR in human and rodents is reported, it remains unclear whether changes in mtDYN directly affect insulin action with the diabetes-susceptible genotype background. Here, we further prove that restoring mitochondrial network by either overexpression of fusion-related (Mnf1/Mnf2) proteins or inhibition of fission-related proteins (Drp1/Fis1) in diabetes-susceptible cybrids, impaired insulin signaling, and GLUT1/GLUT4 translocation to cell membrane can be repaired both in basal condition and in response to insulin stimulation. Furthermore, Mdivi-1, a chemical compound which attenuates mitochondrial fission by selectively blocking GTPase activity of Drp1 [29], reversed mitochondrial genotype-related imbalance of dynamics and ameliorated the impairment of insulin signal transduction in our diabetes-susceptible cybrid cells. Our data support that mitochondrial dynamic proteins per se play a causal role in IR, and the conclusion is partially supported by the previous studies [9, 22], which demonstrated that inhibition of Drp1-dependent mitochondrial fission pathway had therapeutic potential in IR in animal studies.

Besides IR, an imbalance of mitochondrial networks in neurons favoring mitochondrial fission plays a critical role in the pathogenesis of diabetic neuropathy [30, 31]. Mitochondrial fission also occurs in the kidneys and heart after acute ischemia/reperfusion injury in mice, and prevention of this process is beneficial [32, 33]. Moreover, preserving mitochondrial dynamics protected beta-cells from glucose and palmitic acid-induced pancreatic beta-cell mitochondrial fragmentation and apoptosis [23, 34]. Genetic or pharmacological activation of Akt protected the heart against acute ischemia injury is proved by the effect of Akt-mediated mitochondrial elongation [35]. Cardiac mitochondria have been recognized to play an important role in diabetic cardiomyopathy, heart failure, and pulmonary hypertension. The potential of mitochondrial dynamics as therapeutic targets in tackling cardiovascular disease has been reviewed [36]. Therefore, Mdivi-1 may provide the potential for therapeutic use not only in IR but also in diabetes-related target organ damage. However, there are still many challenges remained to be addressed before it might be applied clinically. Its potential side effects include arrhythmia and cytotoxic to nontarget tissue [37].

Of note, a mutual interaction between mtROS and mtDYN is demonstrated in our studies. In our previous studies, cybrid harboring diabetes-susceptible mitochondrial haplogroup B4 showed fragmented mitochondrial morphology, increased ROS, IR, and mitochondrial dysfunction. All the defects can be improved by antioxidant agent N-acetylcysteine (NAC) [16, 17] that is manipulating ROS, which can alter the dynamics of mitochondria. In the current study, we further prove that manipulating dynamics of mitochondria can alter mtROS. Therefore, the causal role of mtDYN in regulating IR is probably linked through mtROS.

In conclusion, mtDYN play a causal role in diabetes-susceptible mitochondrial haplogroup-related IR. Direct intervention to correct the imbalance of mtDYN, especially reversing profission, provides a novel therapeutic strategy for IR and T2D.

## Data Availability

All the data supporting the results were shown in the paper and can be applicable to the corresponding author.

## Abbreviations

Cybrid: Cytoplasmic hybrid
DAPI: 4′,6-diamidino-2-phenylindole
DMEM: Dulbecco's modified eagle medium
DRP1: Dynamin-related protein 1
FIS1: Mitochondrial fission 1 protein
GLUT1/GLUT4: Glut1/Glut4 glucose transporters
H_2_O_2_: Hydrogen peroxide
IR: Insulin resistance
IRS1: Insulin receptor substrate 1
MFN1 and MFN2: Mitofusins 1 and 2
mtDNA: Mitochondrial DNA
NAC: N-acetyl-L-cysteine
OCR: Oxygen consumption rate
OPA1: Optic atrophy gene 1
PI3K: Phosphatidylinositol 3 (PI3)-kinase
ROS: Reactive oxygen species
SE: Standard error
T2DM: Type 2 diabetes.
